# Comparison of *Bacillus subtilis* transcriptome profiles from two separate missions to the International Space Station

**DOI:** 10.1038/s41526-018-0061-0

**Published:** 2019-01-07

**Authors:** Michael D. Morrison, Patricia Fajardo-Cavazos, Wayne L. Nicholson

**Affiliations:** Department of Microbiology and Cell Science, University of Florida, Merritt Island, FL USA

## Abstract

The human spaceflight environment is notable for the unique factor of microgravity, which exerts numerous physiologic effects on macroscopic organisms, but how this environment may affect single-celled microbes is less clear. In an effort to understand how the microbial transcriptome responds to the unique environment of spaceflight, the model Gram-positive bacterium *Bacillus subtilis* was flown on two separate missions to the International Space Station in experiments dubbed BRIC-21 and BRIC-23. Cells were grown to late-exponential/early stationary phase, frozen, then returned to Earth for RNA-seq analysis in parallel with matched ground control samples. A total of 91 genes were significantly differentially expressed in both experiments; 55 exhibiting higher transcript levels in flight samples and 36 showing higher transcript levels in ground control samples. Genes upregulated in flight samples notably included those involved in biofilm formation, biotin and arginine biosynthesis, siderophores, manganese transport, toxin production and resistance, and sporulation inhibition. Genes preferentially upregulated in ground control samples notably included those responding to oxygen limitation, e.g., fermentation, anaerobic respiration, subtilosin biosynthesis, and anaerobic regulatory genes. The results indicated differences in oxygen availability between flight and ground control samples, likely due to differences in cell sedimentation and the toroidal shape assumed by the liquid cultures in microgravity.

## Introduction

In certain respects, human spaceflight habitats resemble other confined built environments, such as submersible vehicles, aircraft, hospital isolation wards, or remote research installations.^1^ However, the spaceflight environment is unique because it contains two additional altered physical parameters: reduced (micro-)gravity and increased ionizing radiation from solar and galactic sources. Extensive investigations conducted in spaceflight on macroscopic organisms have resulted in a relatively good understanding of the biological effects of microgravity and radiation at levels ranging from the whole body down to the organ, cellular, and molecular level in humans,^2^ animals,^3^ and plants.^4^ While microorganisms have also been the subject of focused research in the spaceflight environment, it has proven more difficult to understand their responses to spaceflight stress.^5–7^ From a theoretical perspective, exposure to microgravity results in a number of alterations in a microbial cell’s immediate surroundings, such as loss of convective mass and heat transfer, reduction in mechanical shear forces, and alterations in the way liquids behave at air and solid interfaces. Changes in such fundamental physical forces alter the rates at which gases, nutrients, signaling molecules, and waste products are exchanged between microbes and their surroundings. It has been proposed that upon perception of these alterations in their environment, microbes mount a complex set of stress responses (the so-called “spaceflight syndrome”^8^).

Considerable effort has been expended to understand microbial responses to spaceflight and their underlying causes. In early studies, various phenotypic outputs from microbes grown in space were measured, such as: growth rate and yield; virulence; biofilm formation and architecture; and resistance to antibiotics or abiotic stresses.^5,6,9^ More recent efforts tended toward gene expression studies using genome-wide techniques such as microarrays to understand how the global pattern of RNA synthesis (i.e., the transcriptome) responds to the spaceflight environment. To date, microarray studies have reported a wide range of responses to spaceflight including increased transcription of genes encoding general metabolism,^10,11^ secondary metabolite biosynthesis,^12^ synthesis of ribosomal proteins,^11,13^ and virulence factors.^13,14^ Regardless of the output measured, it has proven difficult to derive consistent conclusions from these disparate studies due to several confounding factors.

First, until recently spaceflight transcriptome studies have been performed on only a small selection of Gram-negative bacteria (*Salmonella enterica* serovar Typhimurium, *Pseudomonas aeruginosa, Rhodospirillum rubrum*), limiting the ability to generalize conclusions to a broader range of microbes. Second, spaceflight experiments have been conducted under widely different: (i) culture conditions (e.g., media formulations, agar vs. liquid, aeration, temperature); (ii) growth stage of the cultures at harvest; (iii) spaceflight hardware employed; (iv) pre- and post-flight treatment of samples; and (v) types of assays conducted.^6,7^ Third, experimental variation derives from the measurements themselves (technical effects) or from the natural variation inherent in biological systems (biological effects).^15^ In an effort to control for variation, prior microbial spaceflight experiments have included multiple replicates; however, most experiments reported in the literature have been flown on a single mission only. Intense competition for limited cargo space destined to research platforms such as the International Space Station (ISS) generally results in the choice a new experiment taking precedence over a repetition of a previously flown experiment. Because of this, the intrinsic mission-to-mission variability in the response of microbiological systems to the spaceflight environment has remained largely unexplored.

In 2015, we were afforded the opportunity to send an experimental package to the ISS to test the responses of the Gram-positive bacterium *Bacillus subtilis* to the human spaceflight environment. This was the 21st mission to the ISS using Biological Research in Canister-Petri Dish Fixation Unit (BRIC-PDFU) hardware, and the experiment was dubbed BRIC-21. From the BRIC-21 experiment we have previously reported in detail measurements of the growth, antibiotic resistance, frequency and spectrum of mutagenesis exhibited by *B. subtilis* flight (FL) samples in comparison to matched ground control (GC) samples.^16,17^ In addition, we also performed RNA-seq analyses to compare the transcriptomes of BRIC-21 FL vs. GC samples, as we will report in this communication. In 2016 we had the good fortune, in collaboration with the NASA GeneLab group, to fly a second mission to the ISS (dubbed BRIC-23) using the same *B. subtilis* strain, media, and hardware, and again to perform RNA-seq analyses on the samples. Here we provide a comparative analysis of the *B. subtilis* transcriptome profiles from the BRIC-21 and BRIC-23 spaceflight missions. We report on the complete transcriptome profiling of a Gram-positive bacterium grown in the human spaceflight environment (a prior study focused on a subset of primary and secondary metabolite genes in *Streptomyces coelicolor*^12^). In this study, we show the effect of exposure to the human spaceflight environment on the *B. subtilis* transcriptome by identifying sets of genes expressed in common in both the BRIC-21 and BRIC-23 missions.

## Results

RNA-seq was used to characterize the transcriptomic response of *B. subtilis* cultures exposed to the human spaceflight environment of the ISS (FL samples) vs. matched GC samples on two separate missions, BRIC-21 (*n* = 3) and BRIC-23 (*n* = 9). The data were analyzed using the bioinformatics pipeline described in the section 'Methods' and the results are presented below.

### Overview of the datasets

**Fig. 1:**
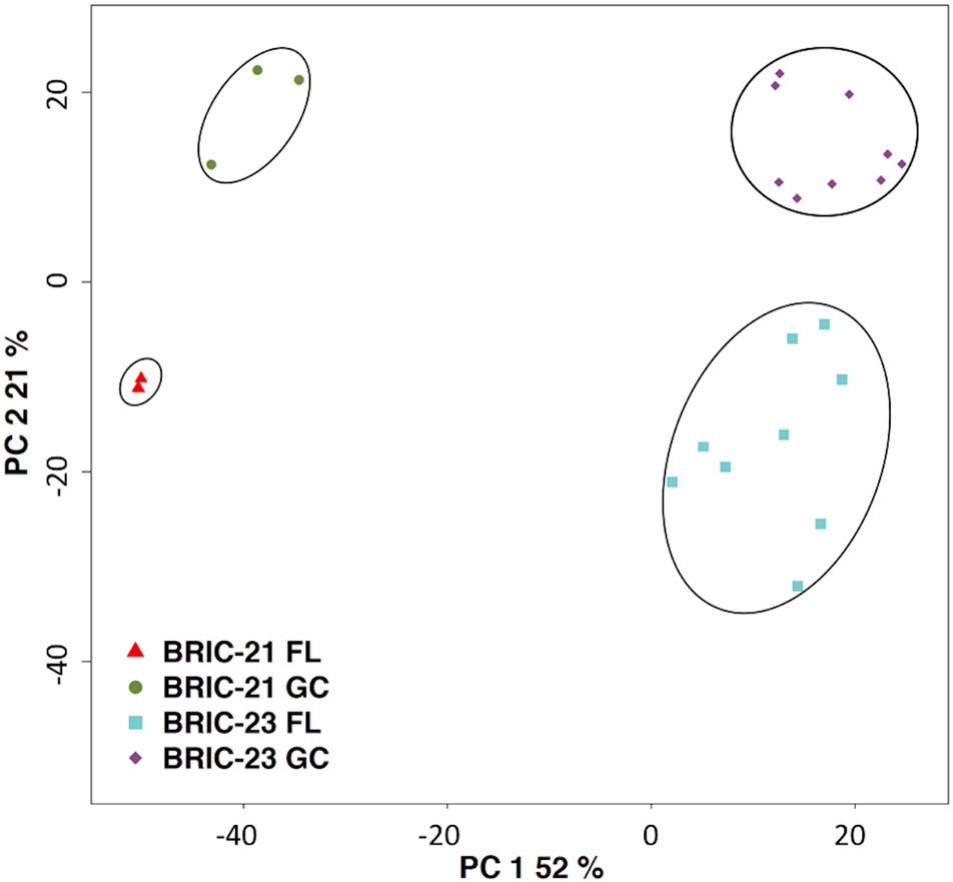
Principal Component Analysis of the datasets from BRIC-21 FL (red triangles) and GC (green circles) samples, and BRIC-23 FL (blue squares) and GC (purple diamonds) samples

In order to assess the quality and reproducibility of the datasets obtained, principal component analysis (PCA) was performed. In the PCA, the first and second principal components explained 52 and 21% of the variance, respectively. Four distinct population clusters were identified corresponding to the four environmental conditions tested (Fig. 1). In the BRIC-21 FL and GC samples, the three replicates were grouped rather tightly, indicating relatively good agreement. In the BRIC-23 FL and GC samples, the nine replicates were somewhat more disperse, but still formed distinct groups (Fig. 1). Examination of Principal Component 1 revealed that the major source of variation in the datasets derived from the differences in the two missions themselves, while variation in Principal Component 2 was due to differences between the FL and GC datasets in each experiment (Fig. 1).

The *B. subtilis* strain 168 transcriptome consists of 4397 total genes, of which 4280 encode proteins.^18^ Analysis of the BRIC-21 RNA-seq data resulted in identification of 293 total genes whose expression differed significantly in FL vs. GC samples, representing ~6.8% of the protein-coding genome. Of these genes, 177 were significantly higher in FL samples, and 116 were significantly higher in GC samples. These data are summarized in Supplemental Table S1. Analysis of the BRIC-23 RNA-seq data resulted in identification of 255 total genes whose expression differed significantly in FL vs. GC samples, representing ~6.0% of the protein-coding transcriptome. Of these genes, 163 were significantly higher in FL samples, and 92 were significantly higher in GC samples. These data are summarized in Supplemental Table S2.

**Fig. 2:**
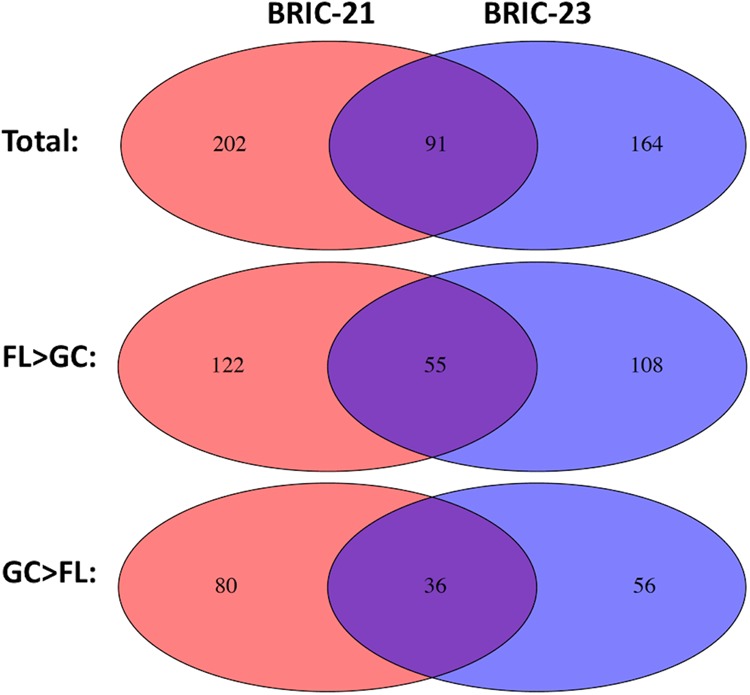
Venn diagrams showing number of genes significantly differentially expressed in the BRIC-21, BRIC-23, or both missions. Total genes (top), genes expressed higher in FL than GC samples (FL > GC; middle) or genes expressed higher in GC than FL samples (GC > FL; bottom) are depicted

We reasoned that comparison of the transcriptome datasets from the BRIC-21 and BRIC-23 experiments would identify genes that were significantly up- or down-regulated in both missions, thus defining genes whose expression was consistently altered in response to spaceflight. A comparison of the datasets obtained from the BRIC-21 and BRIC-23 experiments is depicted graphically as Venn diagrams (Fig. 2). A total of 91 genes were significantly differentially expressed in both experiments. Fifty-five of the shared genes exhibited higher transcript levels in FL samples and 36 genes showed higher transcript levels in GC samples (Fig. 2).

The finding that only ~1/3 of the significantly differentially expressed transcripts were shared in both the BRIC-21 and BRIC-23 missions indicated a substantial amount of between-experiment variation. What could be the source of this variation? While we attempted to keep discrepancies between the two experiments to a minimum, one notable difference between the BRIC-21 and BRIC-23 experiments was the difference in incubation times (25 vs. 36 h), which may have contributed to the ~2/3 discordance in the two datasets. Unfortunately, BRIC-PDFU hardware does not allow direct measurement of growth to be determined in situ during spaceflight. However, because the transcription of genes encoding fundamental growth processes (e.g., replication, transcription, translation) exhibits a positive correlation with growth rate,^19^ as a proxy for growth rate we compared the fold changes for transcripts involved in DNA replication (*dnaE*), RNA transcription (*rpoABCD*), and protein synthesis (*rps* and *rpl* genes encoding small and large ribosomal subunit proteins). Neither limma or DESeq2 identified a significant difference in the transcript expression levels for these genes in either the BRIC-21 or BRIC-23 datasets, suggesting that the FL and GC samples were exhibiting similar growth rates at the time of harvest for RNA extraction.

**Fig. 3:**
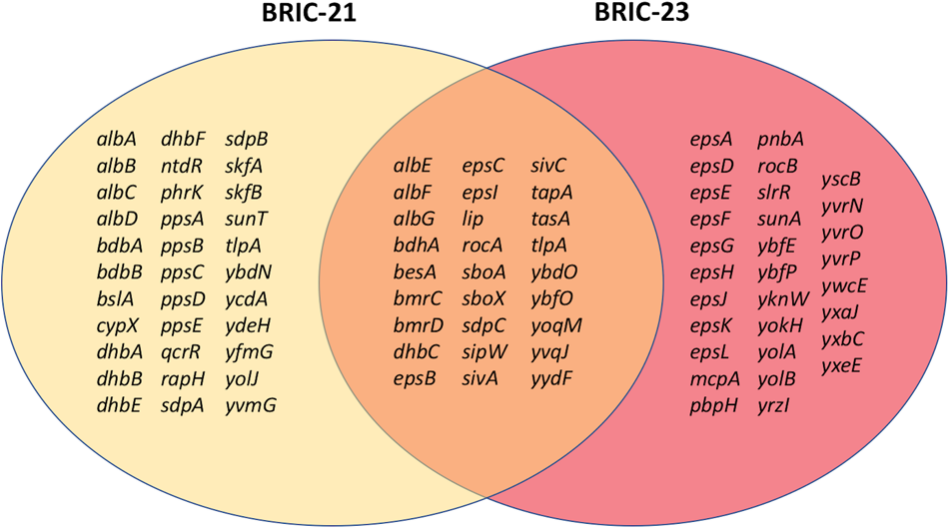
Venn diagram showing genes of the transition-state AbrB regulon significantly differentially expressed in the BRIC-21, BRIC-23, or both missions

In pre-flight ground-based experiments we determined that under ISS ambient temperature (~23 °C), 25 and 36 h of incubation corresponded to late-exponential phase and the “transition state” between exponential and stationary-phase growth, respectively.^20^ Entrance into the transition state in *B. subtilis* results in the transcriptional activation of nearly 300 genes which comprise a regulon under control of the AbrB protein;^21^ we reasoned that comparison of AbrB-controlled transcripts between the BRIC-21 and BRIC-23 datasets might provide insight into the growth phase of these two populations. We found that 60 and 57 AbrB-dependent transcripts were significantly altered in the BRIC-21 and BRIC-23 datasets, respectively, and that 27 transcripts were significantly altered in both experiments (Fig. 3). From this analysis it therefore appeared that cultures in both experiments were at or near the transition phase of growth.

### Genes upregulated in FL samples

The 55 genes whose expression was significantly upregulated in FL samples from both BRIC-21 and BRIC-23 are listed in Table 1. They are arranged according to their BSU locus tag, i.e., in the order that they are located on the *B. subtilis* 168 chromosome map. Examination of the data revealed upregulation of blocks of genes associated with particular phenotypes in *B. subtilis*:

(i) *Biofilm formation*. Under particular environmental conditions, various microorganisms can produce biofilms consisting of cells embedded in a matrix of extracellular polymeric substances (EPS) consisting of polysaccharides, proteins, nucleic acids, and lipids.^22^ It has been reported that spaceflight exposure promoted biofilm formation in *Pseudomonas aeruginosa*^23^ and *Escherichia coli*,^24^ and promoted invasive growth of the yeasts *Saccharomyces cerevisiae*^25^ and *Candida albicans*.^26^ Bacterial biofilms have been implicated in contamination and biofouling of potable water systems in long-duration space habitats, and data from spaceflight have documented biofilms containing *Bacillus* spp. in the Space Shuttle water system.^27^ Laboratory strains of *B. subtilis* such as strain 168 and its descendants do not form robust biofilms due to mutations that have accumulated during their domestication,^28^ and biofilm formation was not noted in FL or GC samples from BRIC-21 or BRIC-23. Nonetheless, numerous biofilm-related genes were observed to be significantly upregulated in both BRIC-21 and BRIC-23 FL samples, including genes of the *srfAA-AB-AC-AD-ycxA* operon, which encodes the pathway for production of the cyclic lipopeptide surfactin^29,30^ and genes of the *tapA-sipW-tasA* operon encoding the major protein matrix component of biofilms^31^ (Table 1). *B. subtilis* biofilms also contain as a major component the exopolysaccharide poly-N-acetylglucosamine,^32^ produced in a biosynthetic pathway encoded by the *epsABCDEFGHIJKLMNO* operon. Our analysis found that only the *epsB, epsC*, and *epsI* genes were significantly upregulated in FL samples of both BRIC-21 and BRIC-23 (Table 1). However, closer examination of the datasets revealed that in the BRIC-23 experiment, 12 of the 15 *eps* genes (*epsABCDEFGHIJKL*) were significantly upregulated in FL samples (Supplemental Table S2). In the BRIC-21 experiment, these 12 genes also displayed a significant upregulation in FL samples (*p* < 0.01), but only three of these genes (*epsB*, *espC*, and *epsI*) met our >2-fold change cutoff, while the remaining nine genes exhibited fold changes slightly below the cutoff (Supplemental Table S1). The last three genes in the *eps* operon, *epsM, epsN*, and *epsO*, were found not to be significantly differentially expressed in FL samples from either mission. In addition, examination of the BRIC-21 dataset revealed that the *bslA*, *ycdA*, and *luxS* genes, also involved in swarming motility and biofilm formation,^33,34^ were significantly upregulated in FL samples (Supplemental Table S1).
(ii) *Biotin biosynthesis*. Biotin (vitamin H) is an essential cofactor for enzymes such as acetyl-CoA carboxylase and pyruvate carboxylase, which are important in fatty acid metabolism and central metabolism, respectively.^35^ Production of biotin from pimelic acid is accomplished by the gene products of the *bioWAFDBI* biosynthetic operon. Examination of the data from Table 1 revealed significant upregulation in FL samples of the *bioW, bioA, bioD*, and *bioB* genes in both the BRIC-21 and BRIC-23 missions, as well as upregulation of the *bioY* gene encoding the energy coupling factor (ECF) transporter biotin-specific S-protein (Table 1). In addition, inspection of the BRIC-21 dataset showed significant upregulation in FL samples of the *bioF* and *bioI* biotin biosynthetic genes, the *yuiG (bioYB)* gene encoding a putative second biotin-specific ECF transporter S-protein, the *yhfT* gene whose product is involved in surfactin production, and the *yhfS* encoding a putative acetyl-CoA C-acetyltransferase (Supplemental Table S1); along with the *bio* genes, these additional genes belong to a regulon under control of a repressor named BirA,^36^ thus are likely upregulated as a block.
(iii) *Siderophores*. Iron is an important cofactor for several enzymes, but is only sparingly soluble in most aerobic environments.^37^ To acquire iron, most microorganisms produce and excrete siderophores which bind iron with high affinity for subsequent import by specific transport systems. *B. subtilis* produces the siderophore bacillibactin (2,3-dihydroxybenzoate) encoded by the *besA-dhbACEBF* operon.^38^ FL samples from both BRIC-21 and BRIC-23 were found to significantly upregulate the *besA* and *dhbC* transcripts (Table 1), and subsequent examination of the BRIC-21 data revealed that the entire *besA-dhbACEBF* operon was significantly upregulated in FL samples (Supplementary Table S1). Another siderophore produced by *B. subtilis* called pulcherriminic acid or pulcherrimin is encoded by two small operons, *yvmC-cypX* and *pchR-yvmA*.^39^ The *yvmC* and *pchR* genes were found to be significantly upregulated in FL samples of both BRIC-21 and BRIC-23 (Table 1), and further inspection revealed that both the *yvmC-cypX* and *pchR-yvmA* operons were significantly upregulated in FL samples from BRIC-21 (Supplemental Table S1).
(iv) *Arginine biosynthesis*. The amino acid arginine is produced in a pathway encoded by the *argCJBD-carAB-argF* operon which converts glutamate to citrulline, and the *argGH* operon, which converts citrulline to arginine.^40^ The *argGH* operon was found to be significantly upregulated in both BRIC-21 and BRIC-23 flight samples. Closer examination of the datasets revealed significant upregulation of *argCJBD-carAB-argF* operon transcripts in BRIC-21 FL samples (Supplemental Table S1) and significant upregulation of the arginyl-tRNA genes *trnJ-Arg* and *trnE-Arg* in BRIC-23 FL samples (Supplemental Table S2).
(v) *Manganese transport*. The major manganese ABC-type transporter in *B. subtilis*, encoded by the *mntABCD* operon, was found to be strongly upregulated in both BRIC-21 and BRIC-23 FL samples (Table 1). In addition, closer examination of the datasets revealed that the manganese-proton symporter *mntH* was significantly upregulated in BRIC-23 FL samples (Supplementary Table S2). Paradoxically, in BRIC-21 FL samples we also observed significant upregulation of the *ydfM* gene, encoding a putative Mn(II) efflux pump (Supplementary Table S1).
(vi) *Resistance and toxin genes*. A number of genes encoding resistance and toxic functions were observed to be upregulated in FL samples of both BRIC-21 and BRIC-23, including: *ybfO*, encoding a putative erythromycin esterase; the *bmrCD* operon, encoding a multidrug efflux transporter; *yvqJ*, encoding a putative macrolide-efflux protein; *cadA*, encoding a cadmium efflux pump; and *sdpC*, which encodes a lytic toxin (Table 1). At first glance, increase in the aforementioned transcripts might suggest that FL samples could be exhibiting a higher degree of resistance to antibiotics or toxic compounds. However, in a separate publication we reported that replicate samples from the BRIC-21 experiment were exposed post-flight to a battery of 72 antibiotics and growth inhibitors, and no significant difference in resistance levels was found in FL vs. GC samples.^16^
(vii) *Inhibitors of sporulation initiation*. Sporulation in *B. subtilis* is triggered when cells sense the depletion of nutrients in their environment. Three genes [*sivA, sivB (bslA)*, and *sivC*; *siv* for sporulation-inhibitory vegetative genes] have recently been described which actively inhibit the initiation of sporulation when *B. subtilis* is growing in the presence of sufficient nutrients.^41^ Both the *sivA* and *sivC* genes were found to be significantly upregulated in FL samples of both BRIC-21 and BRIC-23 (Table 1), and as mentioned above, the *sivB* (*bslA*) gene associated with biofilm formation was upregulated in BRIC-21 FL samples (Supplemental Table S1).

**Table 1:** List of genes significantly upregulated in FL samples both in BRIC-21 and BRIC-23 experiments^a^

Locus Tag	Gene Name	BRIC-21		BRIC-23		Annotated function	Regulon
		DESeq2	limma	DESeq2	limma		
BSU02050	*ybdO*	1.94	2.03	1.25	1.24	Unknown	AbrB, SigD
BSU02140	*glpT*	1.43	1.48	1.06	1.10	Glycerol-3-phosphate transporter	GlpP, CcpA, PhoP, SigA
BSU02310	*ybfO*	1.46	1.51	1.89	1.99	Similar to erythromycin esterase	AbrB, SigW
BSU02700	*lipA*	1.44	1.50	1.23	1.27	Extracellular lipase	Unknown
BSU02710	*yczC*	1.93	1.99	1.08	1.07	Unknown	Unknown
BSU03200	*putB*	3.16	3.67	1.02	1.02	Proline dehydrogenase	CodY PutR, SigA, Spo0A
BSU03480	*srfAA*	2.61	2.91	1.84	1.85	Surfactin synthase subunit 1	Abh, CodY, ComA, PerR, PhoP, SigA, Spx
BSU03490	*srfAB*	3.06	3.28	2.19	2.22	Surfactin synthase subunit 2	Abh, CodY, ComA, PerR, PhoP, SigA, Spx
BSU03510	*srfAC*	3.16	3.37	2.47	2.51	Surfactin synthase subunit 3	Abh, CodY, ComA, PerR, PhoP, SigA, Spx
BSU03520	*srfAD*	3.18	3.46	2.63	2.67	Surfactin synthase thioesterase subunit	Abh, CodY, ComA, PerR, PhoP, SigA, Spx
BSU03530	*ycxA*	2.37	2.44	2.08	2.08	Unknown	ComA
BSU_tRNA_36	*trnD-Thr*	1.02	1.13	1.03	1.06	Threonyl transfer RNA	Unknown
BSU09710	*bmrC*	1.01	1.05	1.33	1.33	Multidrug resistance ABC transporter ATP-binding protein	AbrB, BmrB
BSU09720	*bmrD*	1.05	1.09	1.21	1.17	Multidrug resistance ABC transporter ATP-binding protein	AbrB, BmrB
BSU10370	*bioY*	3.81	4.10	1.40	1.43	S-protein of biotin ECF transporter	BirA
BSU11381	*appA/1*	2.36	2.45	1.31	1.30	Oligopeptide ABC transporter, inactive pseudogene in strain 168	CodY, ScoC, TnrA
BSU11382	*appA/2*	2.52	2.61	1.17	1.10	Oligopeptide ABC transporter, inactive pseudogene in strain 168	CodY, ScoC, TnrA
BSU12010	*manP*	2.38	2.67	1.38	1.47	PTS system-mannose-specific transporter subunit EIIBCA	ManR, SigA
BSU15960	*sivC*	2.89	3.06	2.67	2.71	Inhibitor of entry into sporulation via KinB or KinC	AbrB, SigD
BSU18000	*citB*	1.83	1.99	1.17	1.18	aconitase	CcpA, CcpC, CitB, CodY, FsrA, SigA
BSU21330	*yomK*	1.47	1.58	1.90	1.93	Unknown	SPβ prophage
BSU21420	*bhlA*	1.09	1.34	−1.05	−1.28	Holin-like protein	SPβ prophage
BSU24620	*tasA*	2.22	2.33	3.11	3.38	major component of biofilm matrix, forms amyloid fibers	AbrB, LutR, RemA, SigA, SinR
BSU24630	*sipW*	2.37	2.51	2.81	3.52	Bifunctional signal peptidase I that controls surface-adhered biofilm formation and processes TasA and TapA	AbrB, LutR, RemA, SigA, SinR
BSU24640	*tapA*	2.77	2.93	2.78	2.96	TasA anchoring/assembly protein	AbrB, LutR, RemA, SigA, SinR
BSU26490	*yrkJ*	1.73	1.85	1.96	1.92	Unknown	Unknown
BSU26500	*yrkI*	2.32	2.50	2.22	2.48	Unknown	Unknown
BSU26510	*yrkH*	2.63	2.80	2.50	2.89	Unknown	Unknown
BSU26530	*yrkF*	3.22	3.62	2.77	3.84	Unknown	Unknown
BSU26540	*yrkE*	3.16	3.47	2.76	3.65	Unknown	Unknown
BSU29440	*argH*	2.57	2.67	1.25	1.22	Argininosuccinate lyase	AhrC
BSU29450	*argG*	2.50	2.61	1.29	1.30	Argininosuccinate synthase	AhrC
BSU30200	*bioB*	4.35	4.64	1.20	1.16	Biotin synthase	BirA
BSU30210	*bioD*	4.77	5.05	1.67	1.73	Dethiobiotin synthase	BirA
BSU30230	*bioA*	4.57	4.75	1.36	1.31	Lysine-8-amino-7-oxononanoate aminotransferase	BirA
BSU30240	*bioW*	5.63	5.94	2.31	2.41	6-carboxyhexanoate–CoA ligase	BirA
BSU30740	*mntD*	3.95	4.11	3.13	3.32	Manganese ABC transporter (permease)	MntR
BSU30750	*mntC*	3.97	4.12	2.98	3.31	Manganese ABC transporter (membrane protein)	MntR
BSU30760	*mntB*	3.83	3.98	2.82	3.15	Manganese ABC transporter (ATP-binding protein)	MntR
BSU30770	*mntA*	3.12	3.32	2.70	2.76	Manganese ABC transporter (Mn-binding lipoprotein)	MntR
BSU31250	*tlpA*	1.42	1.47	1.32	1.33	Methyl-accepting chemotaxis protein	AbrB, SigD
BSU31990	*dhbC*	2.48	2.57	1.05	1.08	Isochorismate synthase; siderophore bacillibactin synthesis	AbrB, Fur, Kre, SigA, SigI
BSU32010	*besA*	2.31	2.51	1.39	1.40	Trilactone hydrolase, catalyzes ferri-bacillibactin hydrolysis leading to cytosolic iron release	AbrB, Fur
BSU32450	*pucL*	1.07	1.32	−1.14	−1.32	Urate oxidase	PucR, SigA, TnrA
BSU33140	*yvqJ*	1.20	1.50	1.29	1.34	Similar to to macrolide-efflux protein	AbrB
BSU33490	*cadA*	2.37	3.38	1.59	2.23	Cadmium transporting ATPase, resistance to cadmium	CzrA, SigA
BSU33770	*sdpC*	4.41	4.61	1.56	1.61	Toxin, collapses the proton motive force and induces autolysis, kills non-sporulating cells, induces activity of SigW	AbrB, Rok, Spo0A
BSU34290	*epsI*	1.01	1.10	1.66	1.68	Glycosyltransferase, synthesis of extracellular poly-N-acetylglucosamine	AbrB, EAR riboswitch, RemA, SigA, SinR
BSU34350	*epsC*	1.12	1.17	1.48	1.48	UDP-sugar epimerase, required for extracellular polysaccharide synthesis	AbrB, EAR riboswitch, RemA, SigA, SinR
BSU34360	*epsB*	1.01	1.04	2.29	2.41	Extracellular polysaccharide synthesis, protein tyrosine kinase	AbrB, EAR riboswitch, RemA, SigA, SinR
BSU35070	*yvmC*	1.93	2.11	1.15	1.13	Cyclodipeptide synthase; biosynthesis of the extracellular iron chelate pulcherrimin	AbrB, PchR
BSU35080	*pchR*	3.70	3.91	1.55	1.53	Transcriptional repressor (MarR family), controls the expression of genes involved in pulcherriminic acid biosynthesis	CcpA, PchR
BSU37780	*rocA*	1.10	1.31	−1.14	−1.35	3-hydroxy-1-pyrroline-5-carboxylate dehydrogenase; arginine, ornithine and citrulline utilization	AbrB, AhrC, CodY, RocR, SigL
BSU37800	*sivA*	1.62	1.79	2.14	2.20	Inhibitor of KinA autophosphorylation, and subsequently of entry into sporulation	AbrB
BSU40180	*yydF*	4.55	4.77	2.51	2.82	Secreted peptide, controls LiaR-LiaS activity	AbrB, Rok, SigA

^a^Values are log_2_-fold FL:GC expression ratios. Gene names, annotated functions, and regulons are from Subtiwiki (http://subtiwiki.uni-goettingen.de/v3/index.php)^78^, accessed on September 24, 2018

### Additional genes upregulated in FL samples

Examination of Table 1 revealed a number of additional genes upregulated in both BRIC-21 and BRIC-23 FL samples for which no clear phenotypic consequence could be discerned. First, a number of genes encoding products of unknown function were upregulated, including *ybdO, yczC, ycxA*, the *yrkEFHIJ* operon (Table 1). Second, genes encoding transporters for glycerol-3-phosphate (*glpT*), mannose (*manP*), and oligopeptides (*appA/1* and *appA/2*) were upregulated, as were genes encoding an extracellular lipase (*lipA*), proline dehydrogenase (*putB*), aconitase (*citB*), a single methyl-accepting chemotaxis protein (*tlpA*), urate oxidase (*pucL*), and a secreted peptide controlling LiaR-LiaS activity (*yydF*) (Table 1). Each of these belong to its own cohort of genes devoted to different functions in *B. subtilis*, but expression of the other members of the cohort were not significantly altered. As an example, the threonyl transfer RNA gene *trnD-Thr* is transcribed as part of a 16-tRNA gene operon located just downstream from the ribosomal RNA *rrnD* gene cluster in *B. subtilis*,^42^ but it is unclear why only this transcript, and not the entire operon, was significantly upregulated. Third, two genes encoded by the prophage SPβ (*yomK* and *bhlA*) were upregulated in FL samples (Table 1), but numerous SPβ-related genes were also upregulated in GC samples (Table 2), so no coherent pattern of gene expression could be ascertained.

**Table 2:** List of genes significantly upregulated in GC samples both in BRIC-21 and BRIC-23 experiments^a^

Locus Tag	Gene Name	BRIC-21		BRIC-23		Function	Regulon
		DESeq2	limma	DESeq2	limma		
BSU03050	*ldh*	3.26	3.61	1.52	2.31	L-lactate dehydrogenase	Rex, SigA
BSU03060	*lctP*	3.18	3.35	1.18	1.78	L-lactate permease	Rex, SigA
BSU03290	*nasE*	1.63	1.66	1.12	1.20	Assimilatory nitrite reductase (subunit)	Fur, NsrR, ResD, SigA, TnrA
BSU03300	*nasD*	2.45	2.66	1.42	2.02	Assimilatory nitrite reductase (subunit)	Fur, NsrR, ResD, SigA, TnrA
BSU05720	*ydhE*	1.32	1.33	1.23	1.27	Similar to macrolide glycosyltransferase	LiaR
BSU06240	*bdhA*	1.45	1.46	1.19	1.32	Acetoin reductase/butanediol dehydrogenase	AbrB
BSU10230	*yhfH*	1.19	1.29	1.59	1.74	Unknown	Unknown
BSU17710	*tatAC*	1.30	1.33	1.28	1.33	Component of the twin-arginine translocation pathway	Unknown
BSU19180	*des*	1.07	1.07	1.23	1.35	Phosphlipid desaturase	DesR, SigA
BSU19190	*desK*	1.08	1.07	1.72	1.85	Two-component sensor kinase, regulation of cold shock expression of *des*	DesR, SigA
BSU20580	*yoqM*	1.90	1.94	1.80	2.18	Unknown	SPβ prophage
BSU20760	*yopU*	1.10	1.15	1.29	1.73	Unknown	SPβ prophage
BSU20770	*yopT*	1.10	1.14	1.00	1.11	Unknown	SPβ prophage
BSU21050	*yonN*	1.07	1.10	1.27	1.42	DNA-binding protein HU 2	SPβ prophage
BSU21320	*yomL*	1.59	1.64	1.24	1.54	Unknown	SPβ prophage
BSU21329	*youB*	1.52	1.57	1.16	1.30	Unknown	SPβ prophage
BSU29310	*cmoJ*	1.02	1.12	1.14	1.43	Alkyl monooxygenase, required for the conversion of S-methyl-cysteine to cysteine	AscR, CymR, SigA
BSU29340	*tcyN*	1.42	1.55	1.17	1.37	Cystine ABC transporter (ATP-binding protein)	AscR, CymR, SigA
BSU29360	*tcyL*	1.33	1.50	1.06	1.21	Cystine ABC transporter (permease)	AscR, CymR, SigA
BSU30660	*ytkA*	1.58	1.60	1.35	1.43	Unknown	unknown
BSU37250	*narI*	2.58	2.67	1.42	2.03	Nitrate reductase (gamma subunit)	Fnr, SigA
BSU37260	*narJ*	2.86	3.01	1.36	2.15	Chaperone for the nitrate reductase (protein J)	Fnr, SigA
BSU37270	*narH*	3.15	3.32	1.28	2.08	Nitrate reductase (beta subunit)	Fnr, SigA
BSU37280	*narG*	3.24	3.41	1.17	1.95	Nitrate reductase (alpha subunit)	Fnr, SigA
BSU37310	*fnr*	2.14	2.20	1.56	1.92	Transcriptional regulator of anaerobice genes	Fnr, NsrR, ResD, SigA
BSU37320	*narK*	3.03	3.16	1.32	1.90	Nitrite extrusion protein	Fnr, NsrR, SigA
BSU37350	*sboA*	2.91	3.17	1.48	1.88	Subtilosin-A	AbrB, ResD, Rok, SigA
BSU37360	*sboX*	2.70	3.31	1.38	1.63	Bacteriocin-like product	AbrB, ResD, Rok, SigA
BSU37410	*albE*	1.87	2.29	1.17	1.22	Antilisterial bacteriocin (subtilosin) production	AbrB, ResD, Rok, SigA
BSU37420	*albF*	2.07	2.61	1.42	1.52	Antilisterial bacteriocin (subtilosin) production	AbrB, ResD, Rok, SigA
BSU37430	*albG*	2.44	2.57	1.32	1.40	Antilisterial bacteriocin (subtilosin) production	AbrB, ResD, Rok, SigA
BSU37440	*ywhL*	2.13	2.77	1.35	1.39	Unknown	unknown
BSU38060	*ywcJ*	3.09	3.28	1.87	2.06	Similar to nitrite transporter	Rex
BSU38070	*sacT*	1.60	1.66	1.12	1.16	Transcriptional antiterminator for the *sacP-sacA-ywdA* operon	DnaA, SacT
BSU38730	*cydD*	3.03	3.27	1.52	2.08	ABC transporter required for expression of cytochrome bd (ATP-binding protein)	CcpA, Rex, ResD, SigF
BSU38740	*cydC*	3.27	3.51	1.26	1.89	ABC transporter required for expression of cytochrome bd (ATP-binding protein)	CcpA, Rex, ResD, SigF

^a^Values are log_2_-fold GC:FL expression ratios. Gene names, annotated functions, and regulons are from Subtiwiki (http://subtiwiki.uni-goettingen.de/v3/index.php),^78^ accessed on September 24, 2018

### Genes upregulated in GC samples

The 36 genes whose expression was significantly upregulated in GC samples from both BRIC-21 and BRIC-23 are listed in Table 2. They are arranged according to their BSU locus tag, i.e., in the order that they are located on the *B. subtilis* 168 chromosome. Examination of the data revealed upregulation of several blocks of genes associated with the response of *B. subtilis* to oxygen limitation. A previous study^43^ reported that the global response of the *B. subtilis* transcriptome to strict anaerobiosis resulted in the induction or repression of hundreds of genes involved in a variety of cell functions including carbon metabolism, electron transport, iron uptake, antibiotic production, and stress responses. Our data revealed that only a subset of the entire anaerobic regulon was activated, indicating that cells in GC samples underwent only partial oxygen deprivation, and these genes are described below.

(i) *Fermentation*. TSYG medium contains the fermentable sugar glucose, and *B. subtilis* is capable of mixed-acid fermentation using separate pathways for production of the end products acetate, lactate, acetoin, 2,3-butanediol, and ethanol.^44^ In GC samples from both the BRIC-21 and BRIC-23 missions we observed significant upregulation of the *ldh-lctP* operon for fermentation of lactate from pyruvate,^45,46^ as well as the *alsSD* operon for fermentation of acetoin from pyruvate^47^ and the *bdhA* gene for fermentation of 2,3-butanediol from acetoin^48^ (Table 2). Interestingly, we did not note any significant changes in the expression of genes involved in acetate or ethanol fermentation in our samples (Supplemental Tables S1 and S2).
(ii) *Anaerobic respiration*. Growth under oxygen-limiting conditions results in two major modifications of the *B. subtilis* respiratory electron transport chain. First, oxygen depletion activates synthesis of the high-affinity cytochrome *bd* ubiquinol oxidase encoded by the *cydABCD* operon.^49^ We observed that the *cydC* and *cydD* genes were significantly upregulated in GC samples of BRIC-21 and BRIC-23 (Table 2), and further examination of the datasets revealed that the entire *cydABCD* operon was strongly upregulated in GC samples from BRIC-21 (Supplemental Table S1). Second, anaerobiosis results in activation of genes responsible for utilizing nitrate and nitrite as alternative terminal electron acceptors.^44^ In *B. subtilis* GC samples we noted significant upregulation of the *narGHJI* operon encoding nitrate reductase, the *nasDE* operon encoding nitrite reductase, and the *narK-fnr* operon encoding the NarK nitrite extrusion protein and the Fnr regulator of anaerobic gene expression (Table 2). It is interesting to note that the *ywcJ* transcript, encoding a putative nitrite transporter previously identified as a member of the *B. subtilis* anaerobic regulon,^43^ was also upregulated in GC samples. Because TSYG medium does not provide a significant source of nitrate or nitrite, it is likely that although expression of the *nar* and *nas* genes was induced by oxygen limitation, they did not serve a useful physiological function for cells in the GC samples.
(iii) *Subtilosin production*. Further indication for oxygen limitation in BRIC-21 and BRIC-23 GC samples was evidenced by induction of the genes encoding the antilisterial antibiotic subtilosin A, which was previously shown to be induced by anaerobiosis^43^ (Table 2). The pathway for subtilosin A biosynthesis is encoded by the *sboAXalbABCDEFG* operon,^50^ and in both BRIC-21 and BRIC-23 GC samples the *sboA, sboX, albE, albF*, and *albG* transcripts were significantly upregulated (Table 2). Further inspection of the datasets revealed that the entire *sboAXalbABCDEFG* operon was significantly upregulated in BRIC-21 GC samples (Supplemental Table S1).
(iv) *Other genes belonging to the anaerobic regulon*. Two genes encoding products of unknown or only putatively annotated function (*ydhE* and *ytkA*) were seen to be upregulated in GC samples of both BRIC-21 and BRIC-23 (Table 2). Activation of transcripts for these genes under anaerobic conditions was noted previously.^43^
(v) G*enes regulating anaerobiosis*. The response to oxygen limitation in *B. subtilis* has been well studied and is controlled by a complex regulatory hierarchy.^51^ Oxygen limitation is sensed by the membrane sensor kinase ResE and transmitted to the ResD response regulator, encoded by the last two genes of the *resABCDE* operon. ResD activates expression of a large set of genes and operons including its own (*resABCDE*)*, cydABCD, nasBCDEF*, and *sboAXalbABCDEFG*. One of the genes activated by ResD is *fnr*, itself a regulator of anaerobic gene expression. Fnr activates its own expression (*narK-fnr*), as well as that of *narGHJI* and another regulator encoded by the *arfM* gene. In addition, changes in cell physiology associated with the switch to oxygen limitation activate a transcriptional regulator called AlsR, which activates expression of *alsSD* directly^47,52^ and *bdhA* indirectly.^53^ Meanwhile, the regulatory protein Rex senses changes in the NAD^+^/NADH ratio brought on by oxygen limitation and responds by activating a number of genes including the *cydABCD* and *ldh-lctP* operons.^51^ We were prompted by these observations to search for regulators of anaerobic gene expression in the datasets, and found significant upregulation of the *arfM* gene in BRIC-21 (Supplemental Table S1), the *resD* and *resE* genes in BRIC-23 (Supplemental Table S2), and the *narK-fnr* operon in both BRIC-21 and BRIC-23 GC samples (Table 2).
(vi) *Phospholipid desaturase*. Most prokaryotes regulate membrane fluidity in part by controlling the degree of saturation of membrane phospholipids. *B. subtilis* accomplishes this using a fatty acid desaturase encoded by *des*, the first gene in the *des-desKR* operon.^54,55^ Expression of *des* is activated by exposure to low temperature, mediated through a two-component system composed of a membrane-bound sensor kinase and a response regulator encoded by the *desK* and *desR* genes, respectively.^55^ Transcription of *des* and *desK* were seen to be significantly upregulated in GC samples of both BRIC-21 and BRIC-23 (Table 2), and closer examination of the datasets revealed that the entire *des-desKR* operon was upregulated in BRIC-23 GC samples (Supplemental Table S2). While the biological significance of this observation is at present unclear, it is interesting to note that in previous work we reported that *des-desKR* transcription was also activated by exposure of *B. subtilis* to low atmospheric pressure.^56^
(vii) *Metabolism of a cysteine analog*. A pathway in *B. subtilis* was recently described which can convert the cysteine analog S-methyl-cysteine directly to cysteine as a sulfur source, encoded by the *snaA-tcyJKLMN-cmoOIJ-ribR-sndA-ytnM* operon.^57^ We observed that three genes from this operon (*cmoJ, tcyL*, and *tcyN*) were upregulated in GC samples from both BRIC-21 and BRIC-23 (Table 2). Further examination of the datasets revealed additional genes of the operon significantly upregulated in BRIC-21 (*snaA, tcyJ, tcyK, tcyM, cmoI*, and *ribR*) and BRIC-23 (*cmoO, sndA*, and *ytnM*) GC samples (Supplemental Tables S1 and S2).

### Additional genes upregulated in GC samples

Examination of Table 2 revealed a number of genes significantly up-regulated in GC samples from both BRIC-21 and BRIC-23 for which no clear phenotypic consequence could be discerned. First, three genes encoding products of unknown function (*yhfH*, *ytkA*, and *ywhL*) were upregulated in GC samples (Table 2). Second, upregulation of genes encoding a putative macrolide glycosyltransferase (*ydhE*), a component of the twin-arginine translocase (*tatAC*), and a transcriptional antiterminator (*sacT*) (Table 2), but these genes form parts of larger cohorts of genes which were not themselves significantly upregulated. Third, transcripts for a number of genes encoded by the SPβ prophage (*yoqM, yopU, yopT, yonN, yomL*, and *youB*) were induced, the function of most of which are unknown.

## Discussion

In the present study, we analyzed the effects of spaceflight on the *Bacillus subtilis* transcriptome in two separate spaceflight experiments designated BRIC-21 and BRIC-23. This is the first spaceflight bacterial transcriptome reported from a Gram-positive bacterium, and the first transcriptome study of two separate spaceflight missions using the same bacterial strain, growth media, and hardware. The results uncovered several differences between FL and GC samples that were exhibited in both BRIC-21 and BRIC-23 missions.

### Biofilm-associated transcripts

The observation that transcripts associated with biofilm formation were significantly upregulated both in BRIC-21 and BRIC-23 FL samples is in agreement with the results from prior spaceflight experiments using several different microbes indicating enhanced biofilm production in spaceflight.^23–27^ Although the domesticated laboratory strain *B. subtilis* 168 does not form robust biofilms, we propose that biofilm formation in spaceflight-grown *B. subtilis* could readily be studied in detail by using undomesticated biofilm-producing strains such as NCIB 3610 and its derivatives.^58,59^

### Oxygen limitation-associated transcripts

**Fig. 4:**
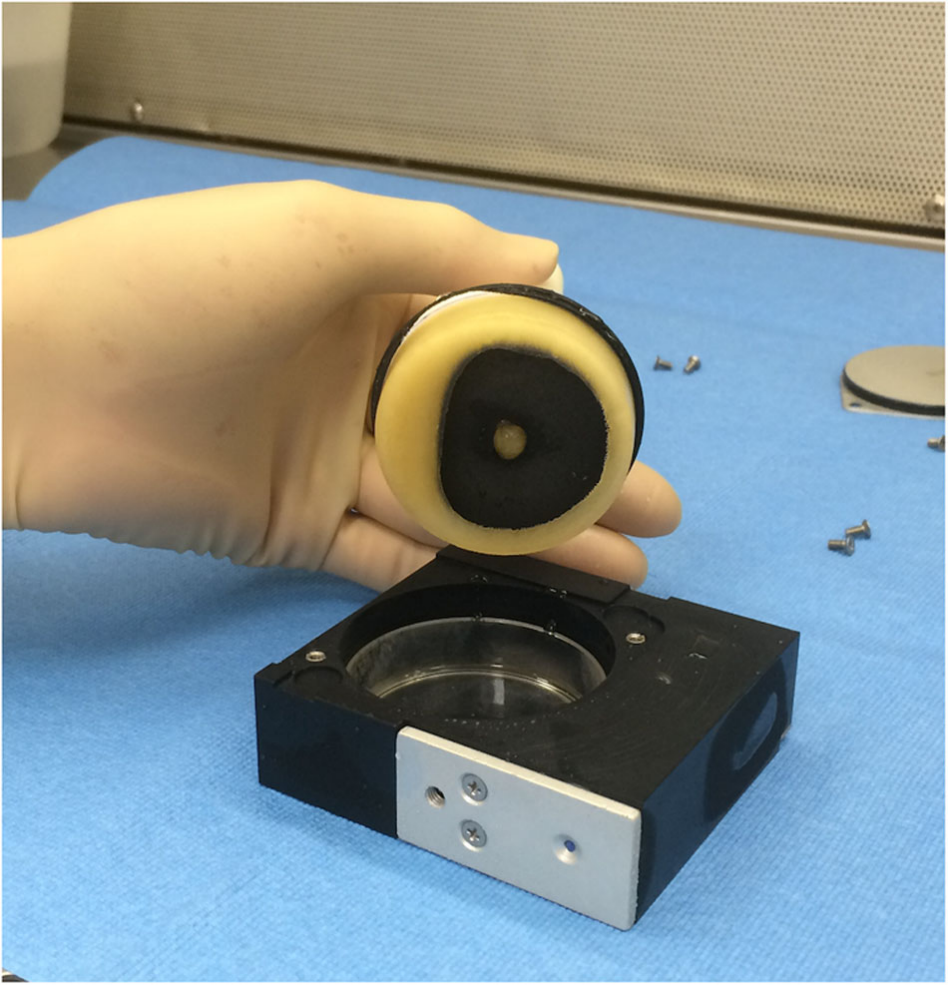
Removal of a typical frozen BRIC-21 FL sample from its PDFU

We noted significant upregulation in GC samples of transcripts associated with fermentation, anaerobic respiration, and subtilosin production, and upregulation in FL samples of transcripts associated with siderophore production. These observations indicate that FL and GC samples were experiencing different degrees of oxygen availability in the two experiments. How could exposure to microgravity vs. 1 x*g* result in different levels of oxygen available to the liquid cultures? First, we noted that during deintegration of frozen BRIC-21 samples from PDFUs, FL samples incubated in microgravity had assumed a toroidal shape (Fig. 4), while GC samples formed a disk-shaped layer in the bottom of the Petri dish as expected. By assuming a toroidal configuration in microgravity, the liquid FL cultures may present a greater surface area to the air phase than in GC samples. Second, under the influence of gravity, cells in GC samples would tend to sediment toward the bottom of the Petri dish, away from the liquid/air interface, thus limiting their access to oxygen. Third, under the influence of gravity, dissolved gases, nutrients, and waste products are transported in liquids by convection and diffusion; in microgravity, convection is negated and transport becomes dominated by diffusive processes.^9^ Oxygen transfer by convection would be expected to be greater in GC than FL cultures, but this effect appears to be outweighed by the first two factors. Unfortunately, because *B. subtilis* prefers an oxygen-rich environment for optimum growth, inclusion of an air-containing headspace in the PDFU cultures is unavoidable. In order to address this hardware limitation, we are currently working on the design and construction of PDFU inserts that will maintain a constant geometry of liquid and air space both in microgravity and in 1-x*g* controls.

### Nutrient utilization-associated transcripts

As stated above, convection ceases in microgravity and the transport of nutrients or waste products through liquid media becomes diffusion-limited.^9^ We observed significantly upregulated transcripts for biotin and arginine biosynthetic genes in FL samples, indicating that availability of these two nutrients differed in FL vs. GC cultures. However, we did not note significant differences between FL and GC samples in the expression of any other biosynthetic pathways, nor was there a significant difference in the final growth yield of cells between FL and GC cultures.^17^ Therefore, it does not appear that exposure to microgravity led to a generalized nutrient deficiency in FL samples.

In our analysis we treated the BRIC-21 and BRIC-23 missions as replicate experiments, but it should be noted that the two experiments differed in incubation times, thus at harvest the BRIC-21 and BRIC-23 samples were at slightly different growth phases (late-exponential and transition-phase, respectively). It is therefore to be expected that the resulting transcriptome profiles would differ somewhat, due to the substantial reorganization of global gene expression which occurs during the transition from exponential to stationary-phase growth in *B. subtilis*.^60^ In addition, it should be kept in mind that measuring the transcriptome captures only one aspect of physiology; it does not take into account regulatory controls exerted at the level of numerous posttranscriptional processes (translation, protein processing and modification, metabolic regulation of enzyme activity, assembly of subcellular structures, etc.) which must take place in order for a microbe to manifest its final phenotype. With these caveats in mind, combining the datasets from the two experiments allowed us to perform a more robust analysis and led to the identification of a common set of genes that were consistently differentially expressed between FL and GC samples in both experiments. These genes, particularly those involved in biofilm formation, will be interesting candidates for future study.

## Methods

### Bacterial strain, media, and growth conditions

The strain used in this study was *Bacillus subtilis* subsp. *subtilis* strain 168 (*trpC2*) from our laboratory stock collection. Medium used throughout was Trypticase Soy Yeast Extract (TSY) medium consisting of (g/L): tryptone, 15; soytone, 5; NaCl, 5; yeast extract, 3; K_2_HPO_4_, 2.5; glucose, 2.5; final pH 7. For semisolid plates, agar was added to TSY to a final concentration of 15.0 g/L. Glycerol was added to TSY liquid medium to 10% (v/v) final concentration, resulting in TSYG medium. *B. subtilis* spores were routinely prepared by cultivation in liquid Schaeffer sporulation medium^61^ at 37 °C with vigorous aeration. The culture was harvested when phase-contrast microscopic examination revealed that it consisted of >90% free spores, usually after 3–4 days of incubation. Spores were purified by lysozyme treatment, buffer washing, and heat shock (80 °C, 10 min) as described previously,^62^ determined by phase-contrast microscopy to be >99% free of cell debris and unsporulated cells, and stored at 4 °C in deionized water. The spore suspension was heat-activated (65 °C, 20 min) before use. From a working suspension (10^8^/mL) of spores in water, aliquots of 0.1 mL (~10^7^ CFU) were applied to the bottoms of sterile 60-mm diameter Petri dishes (Falcon Cat. No. 1007, Fisher Scientific) and air-dried for 48–72 h at room temperature protected from light. Samples were integrated into Biological Research in Canister Dual-Chamber Petri Dish Fixation Units (BRIC-PDFU) spaceflight hardware using aseptic technique as described in detail previously.^20^

### BRIC-PDFU hardware

Biological Research in Canister (BRIC)-Petri Dish Fixation Unit (PDFU) hardware has been described in detail previously.^63,64^ Each BRIC canister enclosed 5 PDFUs, and each PDFU contained a space to accommodate the bottom half of a single 60-mm diameter Petri dish to which air-dried spores were deposited. A separate syringe compartment contained TSYG liquid medium. Growth is initiated by injection of 8.5 mL of TSYG medium into the Petri dish compartment, leaving a headspace of ~18 mL of ambient air which exchanges with the static culture via passive diffusion. The headspace is the only source of additional oxygen, and because the system is hermetically sealed, O_2_ is consumed and CO_2_ accumulates in the culture. Once initiated, there is no mechanism to access cultures for removal of samples during flight.

### Experimental timeline

The schedules of pre-flight, flight, and post-flight activities are described in detail elsewhere for the BRIC-21^16,17^ and BRIC-23^20^ experiments. Experimental details for the two FL experiments were essentially identical in terms of hardware (BRIC-PDFU), inoculum size (10^7^ spores), medium used (TSYG), and media volumes injected (8.5 mL). Slight differences in ambient ISS temperature were recorded inside the BRIC-21 (average ~22.8 °C) and BRIC-23 (average ~22.3 °C) canisters. The only notable difference in the two experiments was the time of incubation for BRIC-21 and BRIC-23 samples, 25 h and 36 h, respectively. In both missions, growth was terminated by transferring BRIC canisters to the −80 °C freezer onboard the ISS. In both cases, samples remained solidly frozen until returned to the lab. Asynchronous GC samples were treated identically to FL samples in terms of hardware, configuration, schedule, temperature profiles, pre- and post-flight handling.^16,17,20^

### BRIC-21 RNA isolation, processing, and sequencing

Frozen FL and GC samples (*n* = 3) were partially thawed at room temperature. Partially thawed samples were transferred into 15-mL conical tubes, and placed on ice until completely thawed. Cells were recovered by centrifugation (7000 × *g*, 20 min, 0 °C) in a benchtop centrifuge. Supernatants were transferred into sterile 15-mL conical tubes and stored at −70 °C. Cell pellets were immediately processed for total RNA extraction and treatment with RNase-free DNase using the RiboPure^TM^ RNA Purification Kit (Thermo Fisher Scientific Inc, Waltham, MA) following the manufacture’s protocols. RNA samples were quantified using a Qubit fluorometer (Invitrogen, Thermo Fisher Scientific Inc, Waltham, MA) and quality evaluated using the RNA 6000 Nano Chip on an Agilent 2100 Bioanalyzer (Agilent Technologies, Santa Clara, CA). RNA Integrity Number (RIN) values ranged from 9.8 to 10.0, indicating high-quality total RNA preparations suitable for further processing. Samples were sent to Hudson Alpha Institute for Biotechnology (Huntsville, AL, USA) for ribosomal RNA depletion, library preparation, and 100-nt paired-end sequencing on an Illumina 2500 instrument.

### BRIC-23 RNA isolation, processing, and sequencing

BRIC-23 sample processing was performed by the GeneLab Sample Processing Lab (NASA Ames Research Center, Mountain View, CA); detailed protocols are described in the BRIC-23 GeneLab dataset GLDS-138 (https://genelab-data.ndc.nasa.gov/genelab/accession/GLDS-138/). Briefly, thawed FL and GC samples (*n* = 9) were centrifuged at 16,000 × *g* for 5 min at 0 °C. Total RNA extraction and RNase-free DNase treatment was also performed using the RiboPure^TM^ RNA Purification Kit (Thermo Fisher Scientific Inc, Waltham, MA) following the manufacture’s protocol. RNA concentrations were measured using a Qubit 3.0 Fluorometer (Thermo Fisher Scientific, Waltham, MA), and RNA quality was assessed on an Agilent 2100 Bioanalyzer (Agilent, Santa Clara, CA). Ribosomal RNA depletion was performed using the Ribo-Zero rRNA Removal Kit for Gram-positive bacteria (Illumina) and sample cDNA libraries were synthesized using KAPA RNA HyperPrep reagents (Roche) and amplified with 10 PCR cycles. Library sequencing was performed on an Illumina HiSeq 4000 platform.

### Read processing, alignment, and quantification

Quality control, mapping, and gene level quantification of Illumina sequences were performed using the Galaxy suite available through the University of Florida’s High-Performance Research Computing Center. The first 12 bases were trimmed off all reads using FASTQ Trimmer v0.014^65^ to remove random hexamer primers, and read quality of the resulting sequences were checked using the FastQC program.^66^ Corresponding paired-end read files were mapped to the *Bacillus subtilis* strain 168 genome [National Center for Biotechnology Information (NCBI) RefSeq accession number NC_000964.3] using Bowtie2 v2.3.2.^67^ Mapping quality was evaluated using SAMStat^68^ followed by gene level quantification using htseq-count v0.6.1.^69^ Gene counts for BRIC-21 and BRIC-23 were separated into separate count matrices for differential expression analysis.

### Differential expression and functional analyses

To reduce false positive results, two Bioconductor packages, limma v3.32.10^70,71^ and DESeq2 v1.16.1,^72^ were used to determine differential expression. These two packages have been shown to exhibit consistent detection precision in comparisons using a large or very small number of replicates^73^ making these packages suitable for our BRIC-21 (*n* = 3) and BRIC-23 (*n* = 9) comparisons. Per limma recommendation, genes with a sum of less than nine counts across all samples were removed before analysis. For limma analyses, filtered genes were normalized between samples using TMM normalization and differential expression analysis was conducted using the built-in voom transformation.^74^ Because DESeq2 requires non-normalized count data, analysis was performed on the raw filtered genes. *P* values from both analyses were corrected for multiple testing using the Benjamini–Hochberg method.^75^ To be considered differentially expressed, genes had to have a >2-fold difference with a *P* value < 0.01 between FL and GC expression using both limma and DESeq2 methods. Genes meeting these criteria were annotated using the NCBI database and the Subtiwiki web server.^76^ Functional analysis was conducted using the Search Tool for the Retrieval of Interacting Genes/Proteins (STRING) protein network database.^77^ STRING protein–protein interaction networks for up- and downregulated genes were generated separately, and Kyoto Encyclopedia of Genes and Genomes (KEGG) pathways were found using the built-in enrichment tools. To check the variability between all the BRIC-21 and BRIC-23 datasets, a PCA was performed in the statistical environment R v3.4.4. Gene loading scores and sample eigenvalues for each principal component were calculated using the *princomp* function in the R package *stats* v3.4.4. Sample eigenvalues for the first two principal components were plotted in R using the default plotting function.

## Electronic supplementary material

Table S1

Table S2

## Data Availability

The RNA-seq datasets have been deposited in the NASA GeneLab Data System under the accession numbers GLDS-185 (BRIC-21) and GLDS-138 (BRIC-23).

## Electronic supplementary material

**Supplementary information** accompanies the paper on the *npj Microgravity* website (10.1038/s41526-018-0061-0).
